# Expression Profiling of Solute Carrier Gene Families at the Blood-CSF Barrier

**DOI:** 10.3389/fphar.2012.00154

**Published:** 2012-08-24

**Authors:** Horace T. B. Ho, Amber Dahlin, Joanne Wang

**Affiliations:** ^1^Department of Pharmaceutics, University of WashingtonSeattle, WA, USA

**Keywords:** choroid plexus, blood-cerebrospinal fluid barrier, BCSFB, solute carriers, CSF, transporters, *Slc* gene, Allen Brain Atlas

## Abstract

The choroid plexus (CP) is a highly vascularized tissue in the brain ventricles and acts as the blood-cerebrospinal fluid (CSF) barrier (BCSFB). A main function of the CP is to secrete CSF, which is accomplished by active transport of small ions and water from the blood side to the CSF side. The CP also supplies the brain with certain nutrients, hormones, and metal ions, while removing metabolites and xenobiotics from the CSF. Numerous membrane transporters are expressed in the CP in order to facilitate the solute exchange between the blood and the CSF. The solute carrier (SLC) superfamily represents a major class of transporters in the CP that constitutes the molecular mechanisms for CP function. Recently, we systematically and quantitatively examined *Slc* gene expression in 20 anatomically comprehensive brain areas in the adult mouse brain using high-quality *in situ* hybridization data generated by the Allen Brain Atlas. Here we focus our analysis on *Slc* gene expression at the BCSFB using previously obtained data. Of the 252 *Slc* genes present in the mouse brain, 202 *Slc* genes were found at detectable levels in the CP. Unsupervised hierarchical cluster analysis showed that the CP *Slc* gene expression pattern is substantially different from the other 19 analyzed brain regions. The majority of the *Slc* genes in the CP are expressed at low to moderate levels, whereas 28 *Slc* genes are present in the CP at the highest levels. These highly expressed *Slc* genes encode transporters involved in CSF secretion, energy production, and transport of nutrients, hormones, neurotransmitters, sulfate, and metal ions. In this review, the functional characteristics and potential importance of these Slc transporters in the CP are discussed, with particular emphasis on their localization and physiological functions at the BCSFB.

## Introduction

The mammalian brain is protected from circulating metabolites, neuroactive substances, drugs, toxins, and blood-borne pathogens by two major barriers: the blood-brain barrier (BBB) and the blood-cerebrospinal fluid (CSF) barrier (BCSFB; Graff and Pollack, 2004; Redzic, 2011). The BBB, formed by brain capillary endothelial cells, is characterized by highly developed tight junctions. The tight junctions of the BBB restrict porous and paracellular pathways of solute diffusion from the blood into the central nervous system (CNS). However, in the choroid plexus (CP), which is a highly vascularized structure located in the four brain ventricles, capillaries are relatively leaky due to a more relaxed tight junction architecture. Hence the barrier function in the CP is provided by the BCSFB, which is formed by a tight monolayer of cuboidal epithelial cells known as choroid plexus epithelial cells (CPEs). The integrity of the BBB and the BCSFB is essential for protecting the brain from the insults of circulating toxins and blood-borne pathogens. Due to their neuroprotective roles, the barrier functions of the BBB and the BCSFB pose a great challenge in delivering many important diagnostic and therapeutic agents to the brain.

This review focuses on the CP, the anatomic locus of the BCSFB. A major function of the CP is to produce and secrete CSF. The tightly sealed cell junctions between the CPEs separate the peripheral blood from the CSF (Spector and Johanson, 1989; Groothuis and Levy, 1997; Segal, 2000; Choudhuri et al., 2003; Zhang et al., 2010). The basolateral membrane of the CP monolayer is in free contact with blood perfusate whereas the apical membrane (also known as the luminal membrane) faces the CSF. The CPEs synthesize the components of the CSF using materials from the circulation and secrete the CSF into the ventricles. Secretion of the CSF is accomplished by active transport of small ions (e.g., Na^+^, Cl^−^, K^+^, and ${\text{HCO}}_{3}^{-}$) and water across the choroidal epithelium. Besides CSF production, the CP may also supply the brain with certain nutrients, hormones, and metal ions while removing metabolites and toxins from the CSF (Miller, 2004; Smith et al., 2004; Redzic et al., 2005). The CP is clinically important as many anatomical and physiological changes occur in this organ following cerebral ischemia and trauma (Ferrand-Drake and Wieloch, 1999; Ferrand-Drake, 2001; Redzic et al., 2005). The CP is also suggested to be a pharmacologically and toxicologically important tissue as it may regulate the distribution of certain drugs and neurotoxic agents between the blood and the CSF (Choudhuri et al., 2003; Smith et al., 2004, 2011; Keep and Smith, 2011).

Owing to their barrier functions, the BBB and the BCSFB express a myriad of distinct membrane transporters to facilitate solute exchange between the CNS and the blood. A major group of membrane transport proteins present at the BBB and the BCSFB belongs to the solute carrier (SLC) superfamily. Currently, more than 370 *SLC* genes have been identified from the human genome and assigned to 51 gene families (Hediger et al., 2004, and the HUGO Gene Nomenclature Committee SLC Family Series)^1^. SLC transporters at the BBB and the BCSFB are vital in maintaining the homeostasis of the CNS via selective and regulated transport of nutrients, hormones, electrolytes, metal ions, and metabolic byproducts at the blood-brain interface. These transporters are also of great pharmaceutical and toxicological significance as they are important determinants of drug disposition and targeting into the brain (Smith et al., 2004; Spector and Johanson, 2006, 2010; Varatharajan and Thomas, 2009; Redzic, 2011). A detailed knowledge of the major SLC transporters expressed at the BBB and the BCSFB is important for the understanding of how various transport systems work at the two CNS barriers to maintain normal functions of the brain. Information on the BBB and the BCSFB transporters also provides insights into the range and characteristics of endogenous compounds and xenobiotics that can traverse the CNS. This knowledge will help to guide rational approaches to target drugs into the brain through endogenous transport systems. In this review, we summarize our current knowledge on the expression, localization, and functional characteristics of transporters encoded by major *Slc* genes found at the BCSFB based upon our recent expression profiling work using high-throughput *in situ* hybridization (ISH) data from the Allen Brain Atlas (ABA).

## Expression Profiling of *Slc* Genes at the Mouse BCSFB

The Allen Brain Atlas^2^ is an open-access genome-wide digital atlas of gene expression in the adult mouse brain developed by the Allen Institute for Brain Science. Using standardized, automated high-throughput ISH procedures, anatomically comprehensive expression patterns of individual genes were obtained at cellular resolution in 56-day-old male C57BL/6J mouse brain (Bhattacharjee, 2006; Lein et al., 2007; Sunkin and Hohmann, 2007). The ABA project has made it possible to examine gene expression in the adult mouse brain at an unprecedented scale and level of resolution. In 2006, we initiated a collaboration with the Allen Institute for Brain Science to systematically analyze mouse *Slc* gene expression using high-quality ISH data generated by the ABA. While the present ABA database has been expanded to ∼20,000 genes, our analysis was primarily based on the dataset released in 2006, which consists of ISH data for over 16,000 genes.

**Figure 1:**
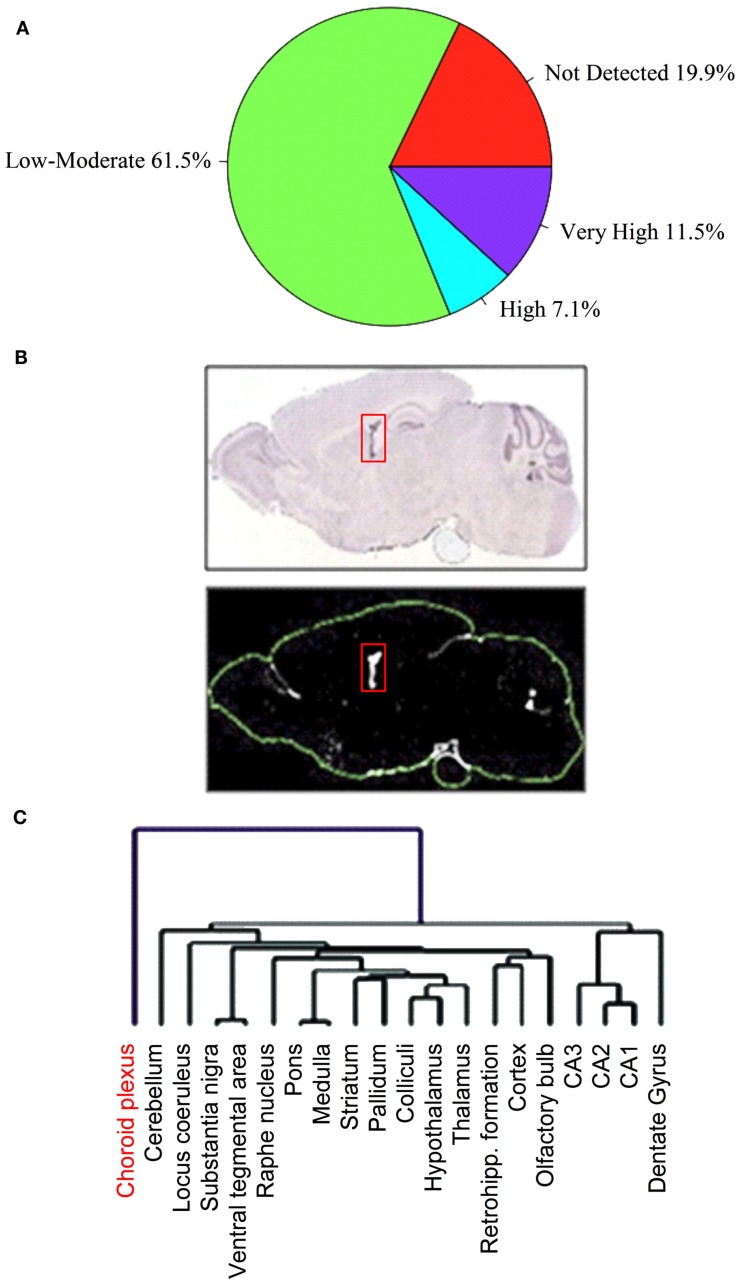
**Distribution of expression profiles for *Slc* genes in the choroid plexus (CP)**. Of the 307 *Slc* genes analyzed in our ABA expression profiling study, 252 *Slc* genes were present in the mouse brain. The pie chart **(A)** shows percentage of these 252 *Slc* genes with expression categories based upon their average expression factor values (*Ê*) in the CP: “not detected” (*E* = 0); “low to moderate” (0 < *E* ≤ 9); “high” (9 < *E* ≤ 13); “very high” (13 < *E* ≤ 20). See Dahlin et al. (2009) for details regarding the definition and calculation of the *E* value. Panels in **(B)** show the restricted localization of *Slc26a11* ISH signals in the CP (structure is indicated by the red boxes). The upper panel represents ISH image and the lower panel is the expression mask generated from the ISH image. In **(C)**, a dendrogram of clustered brain regions (choroid plexus in red) is shown. Reproduced with permission from Dahlin et al. (American Society for Pharmacology and Experimental Therapeutics).

We manually analyzed original ISH photomicrographs for the expression of 307 *Slc* genes, corresponding to ∼82% of presently known mouse *Slc* genes, in 20 anatomically comprehensive brain areas including the BCSFB and the BBB (Dahlin et al., 2009). Of the 307 *Slc* genes analyzed in our study, 252 *Slc* genes were present in the mouse brain, of which ∼80% (202 *Slc* genes) were found at detectable levels in the CP (Figure 1A). The majority of the *Slc* genes in the CP are expressed at low to moderate levels, whereas 28 *Slc* genes are expressed at the highest levels (Figure 1A; Table 1). Most *Slc* genes express in the CP are co-expressed in other brain regions and cell types. However, a very small number of *Slc* genes, such as the sodium-independent sulfate transporter *Slc26a11*, seemed to be expressed only in the choroidal epithelium (Figure 1B). We also compared the *Slc* gene expression patterns in the CP with those in the other 19 brain anatomical areas using unsupervised hierarchical cluster analysis. This analysis, which evaluates the distributional and potential functional relatedness of the *Slc* genes across the brain, generated unique gene expression clusters reflecting the expression relationships of *Slc* gene expression profiles in various brain regions (Figure 1C). The 20 evaluated brain regions formed several subclusters based on correlated *Slc* gene expression patterns (Figure 1C). Strikingly, the expression profile of *Slc* genes in the CP comprises a very distinct cluster outlier, suggesting that there are substantial differences in the types and functions of *Slc* genes in the CP as compared to the other 19 internal structures of the CNS. In these 19 brain areas, anatomically proximal regions were closely co-clustered (Figure 1C), illustrating that functionally and anatomically related structures share a higher degree of overlapping *Slc* gene expression profiles.

**Table 1:** ***Slc* genes with highest expression in the mouse choroid plexus (CP; Dahlin et al., 2009)**.

Gene symbol	Protein name	Substrates	Membrane localization
*Slc2a12*	GLUT12	Glucose	Unknown
*Slc4a2*	AE2	Cl^−^, ${\text{HCO}}_{3}^{-}$	Basolateral
*Slc4a10*	NBCn2, NCBE	Na^+^, ${\text{HCO}}_{3}^{-},$ Cl^−^	Basolateral
*Slc5a3*	SMIT1, SMIT	Myo-inositol	Basolateral
*Slc6a20*	SIT1	Imino acids (e.g., proline)	Unknown
*Slc7a4*	CAT-4	Unknown	Unknown
*Slc7a6*	y^+^LAT2	Large, neutral l-amino acids, and cationic amino acids (e.g., arginine, glutamine)	Unknown
*Slc12a2*	NKCC1	Na^+^, K^+^, Cl^−^	Apical
*Slc13a4*	SUT-1, NaS2	Sulfate	Unknown
*Slc16a2*	MCT8	T3, T4	Apical
*Slc19a1*	RFC1, RFT	*N*^5^-Methyltetrahydrofolate, thiamine derivatives, methotrexate	Apical
*Slco1c1*	OATP1C1	T4, rT3	Apical/Basolateral
*Slc22a17*	BOIT	Unknown	Unknown
*Slc23a2*	SVCT2	l-ascorbic acid	Basolateral
*Slc24a5*	NCKX5	Na^+^, Ca^2+^, K^+^	Unknown
*Slc25a3*	PHC	Phosphate	Mitochondria
*Slc25a4*	ANT1, AAC1	ADP, ATP	Mitochondria
*Slc25a5*	ANT2, AAC2	ADP, ATP	Mitochondria
*Slc25a11*	OGC	Oxoglutarate, malate	Mitochondria
*Slc25a12*	AGC1	Aspartate, glutamate	Mitochondria
*Slc25a17*	ANC1, PMP34	ATP	Unknown
*Slc26a11*		Sulfate	Unknown
*Slc27a1*	FATP1	Long-chain fatty acids	Unknown
*Slc29a4*	PMAT	Biogenic amines, organic cations, adenosine	Unknown
*Slc31a1*	Ctr1	Copper	Unknown
*Slc38a3*	SN1, SNAT3	System N amino acids (e.g., glutamine, histidine, and asparagine)	Apical
*Slc39a4*	ZIP4	Zinc	Unknown
*Slc39a12*		Zinc?	Unknown

## *Slc* Genes Highly Expressed at the Mouse BCSFB

**Figure 2:**
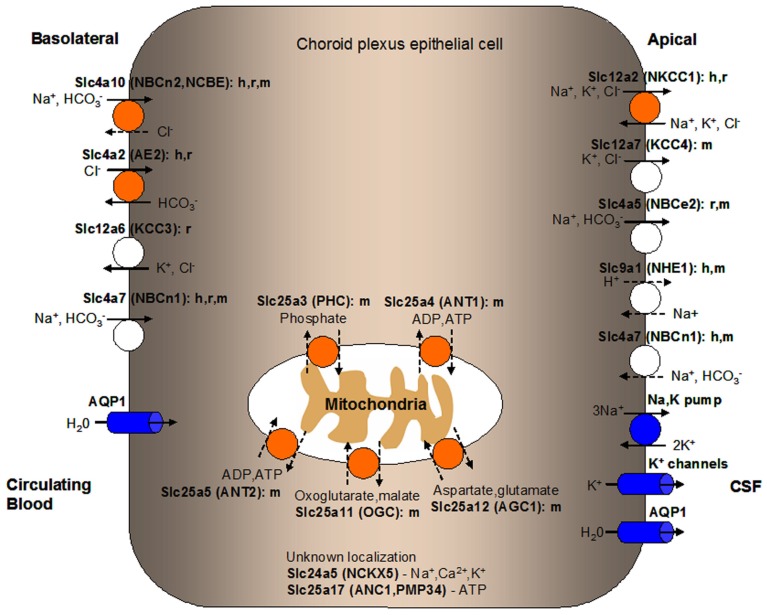
**Localization of solute carrier (SLC) family transporters in the mammalian blood-CSF barrier/choroid plexus (CP) important for the production and secretion of CSF**. Transporters colored in orange are discussed in details in this manuscript. Transporters known to play a role in ion transport at the BCSFB but are not discussed in this review are colored in white. Ion pumps and channels important in mammalian CP function are colored in blue. The suggested membrane localization of these transporters are based on *in vitro*, *ex vivo*, or *in vivo* studies using cells or tissues from various mammalian species as indicated (h, human; r, rat; m, mouse). Ions or substrates being transported by each transporter or channel are shown. Relatively well-established directionality of transport is shown by solid arrows while dashed arrows indicate potential directionality of transport. Transporters with unknown CP localization are listed along with their main substrates. Predicted localization of mitochondrial carriers is shown along with potential physiological substrates. CSF, cerebrospinal fluid.

**Figure 3:**
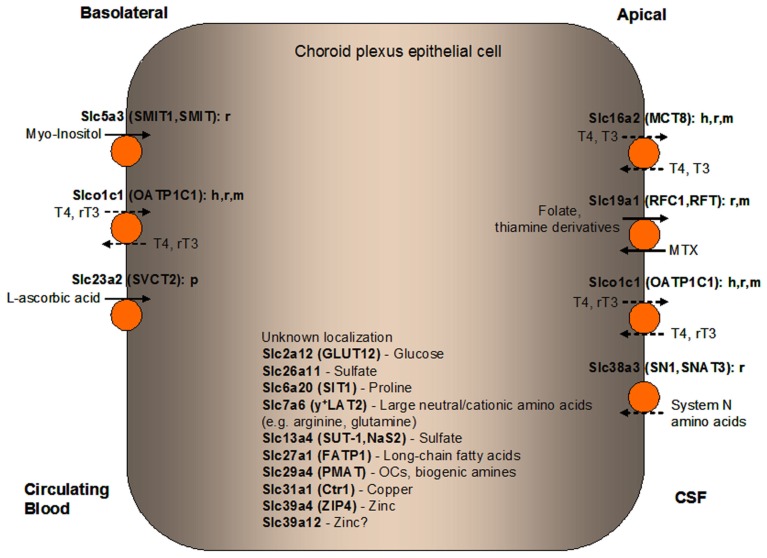
**Localization of solute carrier (SLC) family transporters in the mammalian blood-CSF barrier/choroid plexus (CP) suggested to have a role in nourishing the CP and the brain with amino acids, carbohydrates, fatty acids, vitamins, hormones, monoamines, sulfate, and metal ions**. Transporters colored in orange are discussed in details in this manuscript. The suggested membrane localization of these transporters are based on *in vitro*, *ex vivo*, or *in vivo* studies using cells or tissues from various mammalian species as indicated (h, human; r, rat; m, mouse; p, porcine). Major classes or representatives of substrates for each transporter are shown (T3, triiodothyronine; rT3, reverse triiodothyronine; T4, thyroxine; MTX, methotrexate; OCs, organic cations). Relatively well-established directionality of transport is shown by solid arrows while dashed arrows indicate potential directionality of transport. Transporters with unknown CP localization are listed along with their main substrates. CSF, cerebrospinal fluid.

In our ABA expression profiling study, 202 *Slc* genes were found at detectable levels in the mouse CP (Dahlin et al., 2009). Since it is beyond the scope of this review to discuss every single *Slc* gene expressed in the CP, we limit our discussion to the 28 *Slc* genes found to be most highly expressed in the mouse CP in our ABA expression profile study (Table 1). These *Slc* genes encode several electrolyte transporters involved in CSF secretion (Figure 2) as well as transporters for the transport of nutrients, thyroid hormones, biogenic amines, sulfate, and metal ions (Figure 3). A number of genes from the *Slc25* family of mitochondrial carriers are also highly expressed in the CP, consistent with previous observations that there are mitochondrial enrichment and high Na^+^/K^+^-ATPase activity in the CP (Keep et al., 1986; Keep and Jones, 1990; Cornford et al., 1997; Smith et al., 2004). Two *Slc* genes (*Slc7a4* and *Slc22a17*) appear to be orphan transporters with unknown substrates and are thus not discussed further. Below we will discuss the functional characteristics and potential physiological and pharmacological importance of the transporters encoded by the highly expressed *Slc* genes in the CP, with particular emphasis on their localization and physiological functions at the BCSFB. It should be noted that considerable work has been done previously to characterize a number of Slc members in the CP, including ion transporters (Slc4 and Slc12 families) involved in CSF production (Brown et al., 2004; Praetorius, 2007; Damkier et al., 2010; Majumdar and Bevensee, 2010), oligopeptide transporter 2 (Pept2, Slc15a2; Smith et al., 2004; Kamal et al., 2008), nucleoside transporters (Slc29 family; Markovic et al., 2008), and organic anion and cation transporters (Slc21 and Slc22 families; Ghersi-Egea and Strazielle, 2002; Alebouyeh et al., 2003; Kusuhara and Sugiyama, 2004; Miller, 2004; Zhang et al., 2010; Hosoya and Tachikawa, 2011). Interested readers can refer to related review articles on these transporter families (Table 2).

**Table 2:** **Slc members in the mammalian CP that have been relatively well studied and/or reviewed**.

Slc family/protein	Major substrates	References
Slc4, Slc12	Cl^−^, ${\text{HCO}}_{3}^{-}$ Na^+^, K^+^	Brown et al. (2004), Damkier et al. (2010), Majumdar and Bevensee (2010), Praetorius (2007)
Slc15a2/Pept2	Di-, and tri-peptides	Kamal et al. (2008), Smith et al. (2004)
Slc21, Slc22	Organic anions/cations	Alebouyeh et al. (2003), Ghersi-Egea and Strazielle (2002), Hosoya and Tachikawa (2011), Kusuhara and Sugiyama (2004), Miller (2004), Zhang et al. (2010)
Slc29	Nucleosides/nucleobases	Markovic et al. (2008)
Multiple Slc families		Redzic (2011), Spector and Johanson (2006, 2010)

### Slc transporters involved in ion transport and CSF secretion

A major function of the CP is to secrete CSF, which fills the four brain ventricles, the cranial subarachnoid space, the central canal, and surrounding cavities of the spinal cord. The CSF provides mechanical protection and a stable physiological environment for the CNS. The total CSF volume is ∼140–160 mL in humans and ∼40 μL in mice (Brown et al., 2004; Johanson et al., 2008). The CSF secreted by the CP passes from the lateral ventricles to the third and fourth ventricles, and then to the cranial and spinal subarachnoid spaces, and finally drains into the venous sinus blood via the arachnoid villi (Pardridge, 2011). In humans, the normal CSF turnover rate is about 4–5 times per day (Brown et al., 2004; Johanson et al., 2008). The CSF is not an ultrafiltrate of the plasma (de Rougemont et al., 1960; Davson et al., 1987) and is slightly hypertonic compared to the plasma (Damkier et al., 2010). The overall solute composition of the CSF is similar to that of the blood plasma but the CSF has a much lowered concentration of proteins compared to the plasma (Brown et al., 2004; Redzic and Segal, 2004). The driving force for fluid secretion across the CP epithelium is the active, unidirectional flux of ions from the blood side to the CSF side (e.g., Na^+^, Cl^−^, and ${\text{HCO}}_{3}^{-}$), which creates an osmotic gradient that is accompanied by the movement of water. On the other hand, there is a net movement of K^+^ from the CSF to the blood. There is also a positive electrical potential difference established across the CP epithelium (Damkier et al., 2010).

Since maintaining water volume and electrolyte composition in the CSF is crucial for the CNS to function normally, ion and water transport in the CP need to be tightly regulated. The movement of ions across the CP is mediated by transporters and ion channels differentially expressed at the basolateral (blood-facing) and apical (CSF-facing) membranes of the CPEs (Brown et al., 2004; Redzic and Segal, 2004). The net transport direction of most solutes and water is from the blood to the CSF. Various transporters, ion channels, and aquaporins expressed in the CP mediate CSF secretion and regulate electrolyte composition of the CSF (see Figure 2; Murphy and Johanson, 1990; Speake et al., 2003; Brown et al., 2004; Redzic et al., 2005; Praetorius, 2007; Damkier et al., 2010; Majumdar and Bevensee, 2010). Correspondingly, the highly expressed *Slc* genes in the CP identified in our ABA expression profiling study encode several ion transporters involved in CSF secretion, Slc12a2 (NKCC1), Slc4a2 (AE2), and Slc4a10 [NBCn2, Na^+^-driven Cl^−^-bicarbonate exchanger (NCBE)]. In the following sections, we will discuss the molecular and functional characteristics of these transporters based on published data in the literature. For a more comprehensive review of ion transporters and channels involved in ion transport and CSF secretion across the BCSFB, please refer to Brown et al. (2004), Praetorius (2007), Majumdar and Bevensee (2010), and Damkier et al. (2010).

#### *Slc12a2* (NKCC1)

Our ABA expression profiling study identified high expression levels of *Slc12a2* (Nkcc1) in the mouse CP (Dahlin et al., 2009), which is consistent with previous reports that demonstrated expression of this transporter in the CP in other species (Plotkin et al., 1997a,b; Kanaka et al., 2001; Johanson et al., 2004). NKCC1 was localized to the apical side of the rat and human CP (Plotkin et al., 1997a; Wu et al., 1998; Johanson et al., 2004; Praetorius and Nielsen, 2006), and was suggested to contribute to the regulation of electrolyte concentration in the CSF by mediating the electrically neutral transport of Na^+^, K^+^, and Cl^-^ ions across cell membrane, with a stoichiometry of 1 Na^+^:1 K^+^:2 Cl^-^ (Plotkin et al., 1997a; Wu et al., 1998; Haas and Forbush, 2000). The flux of these ions through the choroidal epithelium is modulated by ion gradients and hormonal activities (Keep et al., 1994). Versatile bidirectional transport via NKCC1 confers flexibility for regulating ion movements and concentrations in the CSF (Johanson et al., 2004).

Studies of the mammalian CP suggest that the key event in CSF secretion is the active, ouabain-sensitive transport of Na^+^ from the epithelial cells to the CSF, which is mediated by the apically positioned Na^+^/K^+^-ATPase (Davson and Segal, 1970; Masuzawa et al., 1984). NKCC1 likely contributes to Na^+^ secretion (supply the CSF with Na^+^) because the apical application of bumetanide (a NKCC inhibitor) inhibits CSF formation (Bairamian et al., 1991; Javaheri and Wagner, 1993; Keep et al., 1994). NKCC1 also likely enriches the CSF with K^+^ to feed the apical Na^+^/K^+^-ATPase (Bairamian et al., 1991; Keep et al., 1994). Alternatively, NKCC1 may take up ions from the CSF as part of regulatory cell volume increase (Wu et al., 1998). The activity of NKCC1 also modulates the level of intracellular Cl^−^ in the CP epithelial cells, helping to maintain cellular volume against changes in extracellular osmolality and intracellular solute content to prevent excessive swelling or shrinkage (Kahle et al., 2008). There is also a growing body of evidence that diuretics which inhibit NKCC1 elicit neuroprotective effects on neurons (Ringel et al., 2000; O’Donnell et al., 2004; Busse et al., 2005). For example, NKCC1 was transiently up-regulated in the CP after traumatic brain injury and bumetanide protected animals from traumatic brain injury-induced edema and neuronal damage (Lu et al., 2006). In diabetes, there is enhanced expression of the NKCC1 cotransporter in the CP for adjusting CSF dynamics and water distribution (Egleton et al., 2003), suggesting compensatory responses at the BCSFB. NKCC1 has also been implicated in Alzheimer’s disease (AD; Johanson et al., 2004). It was suggested that ventriculomegaly and transient elevations in intracranial pressure in AD and normal pressure hydrocephalus may elicit a compensatory response in the CP to downregulate CSF formation by promoting ion reabsorption via the NKCC1 (Johanson et al., 2004). Up-regulated NKCC1 in AD may also help to counter cell shrinkage in the CP (Gosmanov et al., 2003; Johanson et al., 2004), while a rise in CSF K^+^ concentration resulting from neuronal damage could be buffered by the NKCC1 (Keep et al., 1994) and Na^+^ pump in the CP (Johanson et al., 1974). These data implicate the dynamic regulation of NKCC1 under normal and pathophysiological conditions and underscore the importance of NKCC1 in CP and CNS functions.

#### *Slc4a10* (NBCn2, NCBE)

*Slc4a10* encodes a Na^+^ -${\text{HCO}}_{3}^{-}$ cotransporter highly expressed in the mouse, rat, and human CP, where it has been localized to the basolateral membrane (Praetorius et al., 2004; Bouzinova et al., 2005; Praetorius and Nielsen, 2006; Damkier et al., 2007; Jacobs et al., 2008; Dahlin et al., 2009). The rodent protein product of Slc4a10 also exports Cl^−^, and was therefore named NCBE (Wang et al., 2000; Damkier et al., 2010). However, as the human protein product expressed in oocytes does not export Cl^−^ and functions as an electroneutral Na^+^ -${\text{HCO}}_{3}^{-}$ cotransporter, it was suggested to be named as the second electroneutral Na^+^ -${\text{HCO}}_{3}^{-}$ cotransporter (NBCn2; Parker et al., 2008). Slc4a10 is likely to be the main basolateral uptake mechanism for Na^+^ entry into the CP since the secretion of Na^+^ (proportional to the production of CSF) is not only sensitive to basolateral pH but also toward the ${\text{HCO}}_{3}^{-}$ concentration, while it is inhibited by the basolateral application of the anion exchanger inhibitor 4,4′ diisothiocyanostilbene-2,2′-disulfonic acid (DIDS; Saito and Wright, 1983; Deng and Johanson, 1989; Mayer and Sanders-Bush, 1993; Bouzinova et al., 2005; Praetorius and Nielsen, 2006; Damkier et al., 2010). Further, disruption of Slc4a10 in mice leads to an 80% reduction in brain ventricle size, suggesting that the rate of CSF secretion is greatly decreased (Jacobs et al., 2008). Slc4a10 knockout mice also showed a 70% decrease in Na^+^-dependent ${\text{HCO}}_{3}^{-}$ uptake capacity in isolated CP cells, indicating that this transporter is the major ${\text{HCO}}_{3}^{-}$importer (Jacobs et al., 2008; Damkier et al., 2009). Deletion of Slc4a10 led to altered expression patterns of other CP ion transporters/channels, which presumably promoted epithelial cell survival at the expense of maintaining CSF secretion (Damkier et al., 2009; Damkier and Praetorius, 2012). For example, expression of the aquaporin-1 (AQP1) water channel and the apical Na^+^/K^+^-ATPase, which mediates Na^+^ exit from the CPE cells, was greatly decreased in the Slc4a10 knockout mice (Damkier and Praetorius, 2012). In addition, Slc4a10 deficient mice displayed an increased threshold to experimentally induced seizures, probably due to an abnormal regulation of brain pH in the absence of Slc4a10 (Jacobs et al., 2008). In humans, a mutation in the *SLC4A10* promoter region has been implicated in severe complex partial epilepsy and mental retardation (Gurnett et al., 2008).

#### *Slc4a2* (AE2)

Similar to *Slc4a10*, another member of the bicarbonate cotransporter family, *Slc4a2* (Ae2), is also highly expressed in the mouse CP (Dahlin et al., 2009), and it has been localized to the basolateral membrane of the rat and human CP (Lindsey et al., 1990; Alper et al., 1994; Speake et al., 2003; Alper, 2006; Praetorius and Nielsen, 2006). The epithelial $\text{C}{\text{l}}^{-}∕{\text{HCO}}_{3}^{-}$ exchanger AE2 is a Na^+^-independent anion exchanger (Lindsey et al., 1990) which is also expressed in many different cell types. The main function of AE2 in the CP is to regulate intracellular pH through ${\text{HCO}}_{3}^{-}$ efflux which helps to acidify the cytoplasm. However, AE2 may also provide an important route for Cl^-^ influx across the basolateral membrane of the CP. It is speculated that ${\text{HCO}}_{3}^{-}$ is first accumulated across the basolateral membrane through the action of NaHCO_3_ loaders, and ${\text{HCO}}_{3}^{-}$ is then exchanged for Cl^−^ by AE2 (Damkier et al., 2010). AE2 may also serve cell volume regulatory functions or protect cells against alkalization (Praetorius and Nielsen, 2006). The Ae2 knockout mice died at or before weaning and exhibited achlorhydria and osteopetrosis (Gawenis et al., 2004; Alper, 2009).

#### *Slc24a5* (NCKX5)

Another ion transporter that is highly expressed in the mouse CP is the Na^+^/(Ca^2+^-K^+^) exchanger *Slc24a5* (Nckx5; Dahlin et al., 2009). NCKX5 is a potassium-dependent cation exchanger that affects pigmentation in zebrafish and humans (Lamason et al., 2005; Ginger et al., 2008). This is confirmed by a recent knockout study of Nckx5 in the mouse that showed a phenotype of ocular albinism, microscopic differences in the melanosomes, and paler than normal exposed skin surfaces (Vogel et al., 2008). At present, there is no other expression or functional study of this transporter in the CP. However, as a cation exchanger involving Na^+^, Ca^2+^, and K^+^, it is possible that NCKX5 in the CP may participate in the transport of these ions at the BCSFB.

### Mitochondrial carriers involved in energy production

An interesting finding of our ABA expression profiling study is that a number of mitochondrial carrier family members are highly expressed in the mouse CP (Dahlin et al., 2009). These include *Slc25a3* (phosphate carrier/Phc), *Slc25a4* (adenine nucleotide translocase-1/Ant1/Aac1), *Slc25a5* (adenine nucleotide translocase-2/Ant2/Aac2), *Slc25a11* (oxoglutarate carrier/Ogc), *Slc25a12* (aspartate/glutamate carrier 1/Agc1), and *Slc25a17* (Adenine nucleotide carrier from peroxisomes/Anc1/Pmp34). These mitochondrial carrier family members are important for various mitochondrial functions such as phosphorylation of ADP to ATP, uptake of metabolites, linking the energy metabolism compartmentalized between the mitochondrial matrix and the cytosol, and transportation of intermediates important to the Krebs cycle for cellular energy generation (Palmieri, 2004). In contrast to other Slc25 members, Slc25a17 was suggested to be a non-mitochondrial carrier and may be required for the activation of branched-chain and very-long-chain fatty acids that are oxidized in peroxisomes. Details on the functions and physiological/pathological implications of these carriers have been reviewed by Palmieri (2004). Mitochondrial enrichment has been shown in the CP, which is probably due to increased energy demands to fuel the Na^+^/K^+^-ATPase and maintain ion gradient for CSF production (Keep et al., 1986; Keep and Jones, 1990; Cornford et al., 1997; Smith et al., 2004). To date, no studies have been done on any of these proteins with regard to their specific expression, localization and functions in the CP.

### Amino and imino acid transporters

#### *Slc6a20* (SIT1)

*Slc6a20* (SIT1) is a Na^+^-dependent imino acid (proline) transporter and its high expression in the mouse CP was shown by our ABA expression profiling study while another study revealed strong labeling of Sit1 mRNA in the rat CP (Takanaga et al., 2005; Dahlin et al., 2009). There is currently no information as to whether the SIT1 transporter is localized to the apical or basolateral membrane in the CP. SIT1 may serve a primary role in the Na^+^-dependent transport of l-proline in the CPEs. As proline is required for collagen synthesis (McAnulty and Laurent, 1987), SIT1-mediated proline transport may support collagen synthesis in the CP, which has a higher collagen content than elsewhere in the brain (Takanaga et al., 2005).

#### *Slc38a3* (SN1, SNAT3)

A variety of cells can use glutamine for ATP generation (Spolarics et al., 1991). Thus, glutamine transport might be present in the CP and the capillary endothelial cells to meet the energy requirements of these tissues. In support of this hypothesis, the CP that lines the cerebral ventricles expresses high levels of *Slc38a3* (Sn1; Boulland et al., 2002). SN1 is a System N amino acid transporter so named because its naturally occurring substrates (glutamine, histidine, and asparagine) have nitrogen-containing side groups, and are amino acids that have important roles in the brain (Kilberg et al., 1980; Xiang et al., 2003). Sn1 is present in the rat CP as determined by ISH and ICC studies (Chaudhry et al., 1999; Boulland et al., 2002). Our ABA expression profiling study also showed high expression of Sn1 in the mouse CP (Dahlin et al., 2009). Another member of the *Slc38a* family, *Slc38a5* (Sn2), which shares similar amino acid substrates with Sn1, is also expressed in the CP (supplemental Figure 2 from Dahlin et al., 2009). RT-PCR analysis indicated the presence of Sn1 and Sn2 in freshly isolated rat CP (Xiang et al., 2003). The activities of both SN1 and SN2 are pH and Na^+^-dependent (Chaudhry et al., 1999; Nakanishi et al., 2001). Transport studies in isolated CP and primary cultured CP epithelial cells demonstrated predominant pH-dependent apical uptake of glutamine and histidine, suggesting the presence of Sn1 and Sn2 at the apical membrane in the CP (Xiang et al., 1998, 2003). It is possible that the presence of both transporters enables differential regulation of the clearance of glutamine from the CSF. There is currently no definitive conclusion to the relative roles of Sn1 and Sn2 in CP glutamine transport as there are no specific inhibitors or knockout mice for these transporters.

#### *Slc7a6* (y^+^LAT2)

*Slc7a6* (y^+^LAT2) is a member of the heteromeric amino acid transporter family and our ABA expression profiling study found high expression of y^+^Lat2 in the mouse CP (Dahlin et al., 2009). Currently no other expression or functional studies have been done regarding this transporter in the CP. In the rodent brain, y^+^Lat2 is expressed in both neurons and astrocytes (Broer et al., 2000; Heckel et al., 2003; Bae et al., 2005) and it was suggested to function as a bidirectional arginine/glutamine exchanger (Broer et al., 2000; Dye et al., 2004). y^+^Lat2 may play a role in neurons by taking up glutamine as a precursor for glutamate synthesis as well as regulate arginine homeostasis in astrocytes/neurons which is related to nitric oxide synthesis and oxidative/nitrosative stress under hyperammonemia conditions (Broer et al., 2000; Wagner et al., 2001; Zielinska et al., 2012). It is known that y^+^Lat2 needs to be associated with another membrane glycoprotein Slc3a2 (4F2hc) for insertion into the plasma membrane (Torrents et al., 1998). Given that the expression of 4F2hc is also detected in the mouse CP (supplemental Figure 3 from Dahlin et al., 2009), it will be interesting to see if 4F2hc/y^+^LAT2 may play a role in the mammalian BCSFB in the regulation of neutral/cationic amino acids homeostasis in the brain. More studies are needed to identify the localization and physiological substrates of 4F2hc/y^+^LAT2 in the mammalian CP.

### Carbohydrate transporters

#### *Slc2a12* (GLUT12)

Glucose is the primary metabolic fuel for the mammalian brain and a continuous supply is required to maintain normal CNS function. The BBB plays a predominate role in glucose supply to the brain via facilitative glucose transporters such as Slc2a1 (GLUT-1; Devraj et al., 2011). Indeed, in our ABA expression profiling study, several facilitative glucose transporters (e.g., *Slc2a1* and *Slc2a10*) are highly expressed at the BBB (Dahlin et al., 2009). Interestingly, we identified *Slc2a12* (Glut12) as highly expressed in the mouse CP (Dahlin et al., 2009). Among other facilitative glucose transporter family members examined in the ABA expression profiling study, only *Slc2a3* (Glut3) showed moderate levels of expression in the mouse CP (supplemental Figure 4 from Dahlin et al., 2009). GLUT3 is mainly considered as a neuron-specific glucose transporter in rat and human brain tissues (Maher et al., 1992; Shepherd et al., 1992; Haber et al., 1993; Nagamatsu et al., 1993; McCall et al., 1994). Currently, there is little information available regarding the specific GLUT12 expression or function in the CP. GLUT12 transported glucose when it was expressed in *Xenopus laevis* oocytes (Rogers et al., 2003) but the affinity of GLUT12 for this substrate is not known (Augustin, 2010). Both endogenous and over-expressed GLUT12 proteins localize to intracellular compartments (Golgi network) and the plasma membrane in various cell lines (Rogers et al., 2002; Chandler et al., 2003; Flessner and Moley, 2009). Whether GLUT12 is expressed at the apical or basolateral membrane in the CP, and its role in CP glucose transport and/or consumption, remain to be studied.

#### *Slc5a3* (SMIT1, SMIT)

Myo-Inositol (MI) is a carbohydrate that plays a role in many important aspects of cellular regulation including membrane structure, signal transduction, and osmoregulation (Prpic et al., 1982; Garcia-Perez and Burg, 1991; Handler and Kwon, 1996; Guo et al., 1997). MI is a precursor for a family of signal transduction molecules, phosphatidylinositol, and its derivatives that regulate many cellular functions. MI and its various biochemical derivatives are widely distributed in mammalian tissues and cells (Holub, 1986), wherein levels of MI are much higher than in the plasma and interstitial fluids (Dawson and Freinkel, 1961; Berry et al., 1995). Two Na^+^/MI cotransporters, SMIT1 (*SLC5A3*), and SMIT2 (*SLC5A11*), are important for regulating cellular and tissue inositol levels by coupling inositol transport with the physiological transmembrane Na^+^ ion concentration gradient (Kwon et al., 1992; Guo et al., 1997; Coady et al., 2002). The brain inositol levels are 100-fold greater than those found in the periphery (Palmano et al., 1977; Michaelis et al., 1993). Active brain transport of inositol and its stereoisomers is thought to be mediated by inositol transporters at the BBB and the BCSFB. Strong *Slc5a3* mRNA hybridization signals were seen in the rat CP (Inoue et al., 1996; Guo et al., 1997; Yamashita et al., 1998), and our ABA expression profiling study identified high expression of *Slc5a3* in the mouse CP (Dahlin et al., 2009). Smit1 is located at the basal side of the rat CP and is part of the mechanism that uptakes inositol from the basal surface into the CP both *in vitro* and *in vivo* (Spector and Lorenzo, 1975; Inoue et al., 1996; Hakvoort et al., 1998; Angelow et al., 2004). As a Na^+^ and MI cotransporter, SMIT1 was also suggested to play a role in ionic balance in CP epithelial cells by mediating Na^+^ influx (Angelow et al., 2003). At present it is not known how inositol is transported across the apical side of the CP into the CSF.

Studies with Smit1 knockout mice showed neonatal lethality (Berry et al., 2003; Chau et al., 2005) which could be prevented by prenatal maternal MI supplement (Chau et al., 2005). Brain levels of MI were depleted in Smit1 knockout mice (Berry et al., 2003). In adult Smit1 deficient mice rescued by MI supplement, Smit1 is essential for the development and function of the peripheral nerves (Chau et al., 2005). Defects related to CP and CSF functions in Smit1 knockout mice have yet to be elucidated. Clinically, a significant increase in the level of MI was observed in the CSF in Down syndrome (trisomy 21) patients compared with controls (Shetty et al., 1995). It was hypothesized that disruption of MI homeostasis in Down syndrome may affect the developing brain and thus contribute to the pathogenesis of mental retardation, the most consistent and debilitating feature of the syndrome. The mapping of the human SMIT1 gene onto the long arm of chromosome 21 (Berry et al., 1995) supports the hypothesis that altered MI homeostasis may result from increased transport to the CSF (Guo et al., 1997).

### Fatty acid transporters

#### *Slc27a1* (FATP1)

*Slc27a1* (Fatp1) is a long-chain fatty acid transporter identified as highly expressed in the mouse CP in our ABA expression profiling study (Dahlin et al., 2009). At present, no known function of this transporter has been described in the CP. In adipocytes, some FATP1 translocate to the plasma membrane in response to insulin, whereas the majority of FATP1 remain within intracellular structures and primarily reside in the mitochondria (Stahl et al., 2002; Lobo et al., 2007). In one study, FATP1 was suggested as a long-chain fatty acid transporter in human brain microvessel endothelial cells (HBMEC), and reduction in oleate and linoleic acid transport across HBMEC monolayers was detected following loss of FATP1 expression (Mitchell et al., 2011). The physiological role of FATP1 remains to be determined.

### Vitamin transporters

#### *Slc19a1* (RFC1, RFT)

*Slc19a1*, encoding the reduced folate carrier 1 (Rfc1), is highly expressed in the mouse CP (Dahlin et al., 2009) and is localized to the apical membrane of the mouse and rat CP (Wang et al., 2001; Halwachs et al., 2011; Hinken et al., 2011). RFC1 mediates cellular uptake of folate (vitamin B9) and its derivatives, and prefers reduced folates over non-reduced folates (Goldman, 1971; Zhao et al., 2001b; Hinken et al., 2011). RFC1 is a major route for the transport of folates in mammalian cells, and inactivation of Rfc1 in mice resulted in early embryonic lethality (Zhao et al., 2001a,b). While RFC1 is highly expressed at the apical membrane of the CP, the folate receptor is localized to the basolateral membrane of the CP (Weitman et al., 1992; Spector and Johanson, 2006). RFC1 mediates secretion of reduced folates into the CSF, resulting in four times the plasma folate concentration (Spector, 2009; Spector and Johanson, 2010). RFC1 also plays a critical role in cerebral folate homeostasis. Clinically, slowly progressive neurologic dysfunctions have been described in adults and children with an isolated deficiency of folate in the CSF (Botez et al., 1982; Wevers et al., 1994; Ramaekers et al., 2002, 2003; Blau et al., 2003). In addition to folate and its derivatives, RFC1 also transports methotrexate (MTX), an important antifolate drug, and has been implicated in the first step of the elimination of MTX from the CSF into the blood (Spector and Johanson, 2010; Halwachs et al., 2011). This could be important as neurotoxicity is a common complication in patients who receive systemic or intrathecal MTX therapy, resulting in neurologic disorders such as seizures (Hinken et al., 2011). The phosphate derivatives of thiamine (vitamin B1), including thiamine monophosphate (TMP) and thiamine pyrophosphate (TPP), are also known substrates of RFC1 (Rindi and Laforenza, 2000; Ganapathy et al., 2004). TPP is the major thiamine intracellular metabolite and plays a critical role in oxidative phosphorylation and in the pentose phosphate pathway (Zhao et al., 2002). In the CP, RFC1 was suggested as an important regulator of TMP levels (Tallaksen et al., 1997; Wang et al., 2001; Spector and Johanson, 2007).

#### *Slc23a2* (SVCT2)

Within the CNS, l-ascorbic acid (vitamin C) plays a pivotal role as a cofactor in a number of different enzymatic activities related to neurotransmitter processing and as a neuroprotective antioxidant (Angelow et al., 2003; Salmaso et al., 2009). l-ascorbic acid is also necessary for proper myelination of neurons (Eldridge et al., 1987) and has neuromodulatory effects on synaptic transmission (Grunewald, 1993; Rebec and Pierce, 1994). l-ascorbic acid is not synthesized in the brain and therefore needs to be transported from the blood (Spector and Johanson, 2006). The concentration of l-ascorbic acid in some neurons can reach up to 200-fold higher than that measured in the systemic circulation (Rice, 2000) and specific transport mechanisms are responsible for transporting ascorbic acid from the blood (where its concentration is 30–60 μM) into the CSF (where the concentration is maintained at 200–300 μM; Tallaksen et al., 1992; Reiber et al., 1993; Harrison et al., 2010). The CP is a major site of vitamin transport in the brain (Spector and Johanson, 2006). Evidence that l-ascorbic acid reaches the CSF through the route of the CP followed by slow penetration into the brain has been demonstrated by intravenous and intraventricular injection of [^14^C] l-ascorbic acid in animal model experiments (Hammarstrom, 1966; Spector, 1981). Other studies have shown that l-ascorbic acid is taken up by a mechanism that involves the sodium dependent-vitamin C transporter SVCT2 (Tsukaguchi et al., 1999; Angelow et al., 2003).

Our ABA expression profiling study showed that the mouse CP has high expression of the Na^+^-dependent ascorbic acid transporter *Slc23a2* (Svct2; Dahlin et al., 2009). Other studies have also confirmed the expression of Svct2 in the mouse as well as the rat CP (Tsukaguchi et al., 1999; Garcia Mde et al., 2005; Salmaso et al., 2009). Another study in primary cultured epithelial cells of the porcine CP suggested that SVCT2 is present at the basolateral membrane, where it transports l-ascorbic acid from the blood to the CP (Angelow et al., 2003). Meanwhile, Svct2 KO mice fetuses were shown to have significantly lower l-ascorbic acid levels in various tissues and they died perinatally (Harrison et al., 2010). The essential role of Svct2 in mouse development makes it difficult to study the *in vivo* function of Svct2 in the CP using animal knockout models.

### Thyroid hormone transporters

#### *Slco1c1* (OATP1C1)

*Slco1c1* (Oatp1c1) is highly expressed in the mouse CP (Dahlin et al., 2009) and the expression of its encoded protein has been detected by Western blot analysis in the rat CP (Sugiyama et al., 2003). Oatp1c1 is highly expressed at the basolateral (blood) side of the mouse CP as shown by immunohistochemical (IHC) study (Tohyama et al., 2004) and it may play a role in the thyroid hormone thyroxine (T4) uptake from the blood into the CSF (Tohyama et al., 2004). However, in a number of other studies, OATP1C1 protein expression was observed at both basal and apical cell surfaces in mouse, rat as well as human CPs, albeit with a potential bias toward basal localization (Tohyama et al., 2004; Roberts et al., 2008). Oatp1c1 protein expression was also observed at both basal and apical cell surfaces in fetal rat CP (Grijota-Martinez et al., 2011). Rat Oatp1c1 specifically accepts T4 and its inactive metabolite reverse triiodothyronine (rT3) as substrates, and the transport activity of organic anions by the human ortholog OATP1C1 (or OATP-F) is relatively low (Pizzagalli et al., 2002). T4 and rT3 show the highest transport activity and affinity for rat Oatp1c1 among the known substrates (Sugiyama et al., 2003; Tohyama et al., 2004; Chu et al., 2008). Rodent Oatp1c1 shares an 84% sequence homology with its human counterpart (Sugiyama et al., 2003), and human OATP1C1 was also shown to have high transport activity and affinity for T4 and rT3 (Pizzagalli et al., 2002; van der Deure et al., 2008). T4 and its metabolite rT3 affect diverse biological processes in vertebrates, and are important for the function of various organs through their influences on cellular metabolism (Zhang et al., 2010). To exert their effects in the CNS, free thyroid hormones in the circulating blood have to cross the BBB and the BCSFB (Tohyama et al., 2004). Free T4 can cross the CP from the blood to the brain (Southwell et al., 1993) and one-fifth of the T4 in the brain is thought to originate from the CSF that has passed through the CP (Chanoine et al., 1992). OATP1C1 may act as an uptake system in the CP to supply T4 to ependymal cells and circumventricular organs (Dratman et al., 1991). There is also evidence that OATP1C1 participates in the efflux of T4 and metabolites from the brain and the CP to the blood (Kassem et al., 2007; Zhang et al., 2010).

In addition to Oatp1c1, other organic anion transporter family members that are expressed in the rat CP include Oatp1a5 (Oatp3) and Oatp1a4 (Oatp2; Choudhuri et al., 2003; Ohtsuki et al., 2003). Oatp1a5 is localized to the apical membrane of the rat (Kusuhara et al., 2003; Ohtsuki et al., 2003) and mouse CP (Ohtsuki et al., 2004), while Oatp1a4 is localized to the basolateral side of the rat CP (Gao et al., 1999). Both of these transporters may contribute to thyroid hormone transport at the BCSFB.

#### *Slc16a2* (MCT8)

Besides Oatp1c1, *Slc16a2* (Mct8) from the monocarboxylate transporter family is also highly expressed in the mouse CP (Heuer et al., 2005; Dahlin et al., 2009). While OATP1C1 localizes primarily to the basolateral surface in human and rodent CP epithelial cells (as mentioned above), MCT8 expression is mainly concentrated on the apical (CSF-facing) epithelial cell surface in human, mouse, and rat CP (Heuer et al., 2005; Trajkovic et al., 2007; Roberts et al., 2008; Grijota-Martinez et al., 2011). MCT8 has been identified as an active and specific thyroid hormone transporter and it transports T4 and T3, as opposed to OATP1C1 which does not recognize T3 as a substrate (Suzuki and Abe, 2008; Grijota-Martinez et al., 2011; Lang et al., 2011). The transport of T3 by MCT8 is Na^+^-independent, although T4 transport is decreased in the absence of Na^+^ (Friesema et al., 2003). MCT8 is the only transporter for neuronal T3 uptake that has been identified to date. Studies with Mct8-null mice suggest Mct8 as a key player in T3 transport into the brain while other transporters (such as Oatp1c1) may play a more important role in T4 transport into the brain (Tohyama et al., 2004; Dumitrescu et al., 2006; Trajkovic et al., 2007).

The above studies suggest that MCT8 and OATP1C1 may function together to transport T4 from the blood into the CSF, and to efflux thyroid hormones or their inactive metabolites such as rT3 and T2 from the CSF into the blood (Roberts et al., 2008). It should be noted that MCT8 and OATP1C1 are also expressed at the BBB (Sugiyama et al., 2003; Heuer et al., 2005; Dahlin et al., 2009). Previous studies in rats have indicated that only a fraction of T4 in the brain passes through the BCSFB (Dratman et al., 1991), and therefore it was suggested that the main entry of thyroid hormones into the brain occurs via the BBB (Heuer et al., 2005). Further studies will be needed to clarify the respective roles of the BBB and the BCSFB in regulating the concentrations of thyroid hormones and their metabolites in the mammalian brain.

### Plasma membrane monoamine transporter

#### *Slc29a4* (PMAT)

Plasma membrane monoamine transporter (PMAT) is a relatively new SLC transporter first cloned and characterized in our laboratory (Engel et al., 2004). PMAT mediates electrogenic, Na^+^, and Cl^−^ independent, low affinity and high capacity transport of monoamine neurotransmitters (Engel et al., 2004; Itagaki et al., 2012). While PMAT transports many endogenous amines (e.g., 5-HT, dopamine, norepinephrine, histamine), it strongly prefers 5-HT and dopamine over other amines (Engel et al., 2004; Duan and Wang, 2010). PMAT also transports a variety of structurally diverse xenobiotic organic cations such as the neurotoxin 1-methyl-4-phenylpyridinium (MPP^+^) and therapeutic drugs (e.g., metformin; Engel et al., 2004; Engel and Wang, 2005; Zhou et al., 2007) and shares a large substrate overlap with the polyspecific organic cation transporters (OCTs) in the SLC22 family (Engel and Wang, 2005). In humans, PMAT is most strongly expressed in the brain, but transcripts were also found in other organs such as the kidney, heart, and small intestine (Engel et al., 2004; Zhou et al., 2007; Xia et al., 2009).

In our ABA expression profiling study, Pmat was found to be particularly enriched in the mouse CP (Dahlin et al., 2009). This confirms our earlier study in which mouse Pmat was observed as highly expressed within the ventricular epithelial cells of the CP by RT-PCR, non-radioactive ISH, and IHC methods (Dahlin et al., 2007). Pmat mRNA is also highly expressed in the rat CP as shown by ISH study (Vialou et al., 2007). The membrane localization of PMAT in the CP has not been determined. Functional expression in MDCK cells showed apical localization of PMAT in polarized epithelial cells (Xia et al., 2007, 2009), suggesting that the protein may localize to the apical membrane in the CP. Recently, Okura et al. (2011) examined the *in vivo* function of Pmat in the rat CP. Intracerebroventricular (i.c.v.) administration studies were performed to investigate the transport of [^3^H]MPP^+^ across the BCSFB by measuring the elimination rate of [^3^H]MPP^+^ from the CSF after administration. It was found that [^3^H]MPP^+^ administered i.c.v. was eliminated from the CSF, and the elimination was partially inhibited by 5-HT and dopamine, both of which are known substrates of PMAT (Okura et al., 2011). The marked Pmat mRNA expression in the CP epithelium and the transport properties of MPP^+^ across the BCSFB suggest that Pmat is one of the key transporters mediating MPP^+^ clearance from the CSF. We recently demonstrated that two β-carbolines compounds, harmalan, and norharmanium, are transportable substrates of PMAT using cell toxicity assays (Ho et al., 2011). β-carbolines are considered as natural or environmental analogs of MPP^+^ and have been implicated as environmental risk factors for Parkinson’s disease (Nagatsu, 1997; Storch et al., 2004). Thus, by mediating neurotoxin clearance from the CSF, PMAT in the CP may play a protective role against cationic neurotoxins.

### Sulfate transporters

#### Slc26a11

Our ABA expression profiling study identified a sodium-independent sulfate transporter (*Slc26a11*) highly expressed in the mouse CP, and also revealed that while many *Slc* genes expressed in the CP are co-expressed in other brain regions, *Slc26a11* is selectively expressed only in the choroidal epithelia (Figure 1B; Dahlin et al., 2009). Currently, there is no other expression or functional study of *Slc26a11* in the CP. Slc26a11 is reportedly expressed at high levels in the placenta, kidney, and high endothelial venules (Vincourt et al., 2003). Functional expression studies in culture cells revealed that SLC26A11 is targeted to the cell membrane and exhibits Na^+^-independent sulfate transport activity, and is sensitive to the anion exchanger inhibitor DIDS (Vincourt et al., 2003). It was found that in high endothelial venules endothelial cells (HEVEC), SLC26A11 modulates sulfate incorporation into these cells and could play a major role in sulfation of cell adhesion molecules (Vincourt et al., 2003). Sulfation is important in xenobiotic activation/suppression and in posttranslational modification of secreted or membrane-bound proteins and could modify protein–protein interactions involved in leukocyte adhesion, homeostasis, and receptor-mediated signaling (Nowell and Falany, 2006; Sasaki, 2011). If SLC26A11 regulates sulfate availability in the CSF, it may potentially play a role in sulfation of drugs in the brain and/or in sulfation-related posttranslational modification of proteins that are implicated in numerous physiological and pathological processes (Nowell and Falany, 2006). Additional *in vitro* and *in vivo* studies are warranted to elucidate the function of SLC26A11 in the mammalian CP.

#### *Slc13a4* (SUT-1, NaS2)

*Slc13a4* (Sut-1), another transporter that recognizes sulfate as substrate, was also highly expressed in the mouse CP (Matsumoto et al., 2003; Dahlin et al., 2009). However, there is no functional data on this transporter in the CP at present. Unlike SLC26A11, SUT-1 is Na^+^-dependent and is resistant to DIDS (Girard et al., 1999). SUT-1 is speculated to modulate sulfate incorporation into HEVEC and was also suggested to play a role in sulfate incorporation in the placenta (Girard et al., 1999). It has been suggested that the co-expression in HEVEC of the two functional classes of sulfate transporters, SUT-1 and SLC26A11, may allow efficient sulfate incorporation under various physiological conditions because they have very different modes of regulation (Markovich et al., 1993; Bissig et al., 1994). SUT-1 and SLC26A11 may potentially work coordinately to mediate sulfate flux at the BCSFB and/or provide different modes of sulfate uptake and utilization in CP cells. Future studies are required to study the specific roles of SUT-1 and SLC26A11 in sulfate uptake and transport in the CP.

### Metal transporters

#### Copper transporters

All living organisms require copper for growth and development (Harris, 1991; Bull and Cox, 1994). Equipped with a high redox potential, copper serves as a cofactor for proteins involved in a variety of neurological functions and biological reactions such as free radical eradication and connective tissue formation. Thus, copper homeostasis is critical for cell development and survival but excessive levels are not desirable as copper in excess of cellular needs mediates free radical production and the direct oxidation of lipids, proteins, and DNA (Harris, 1991). Cellular uptake, export, and utilization of copper are regulated to control copper homeostasis (Harris, 1991; Vulpe and Packman, 1995; Gybina and Prohaska, 2006). In complex organisms such as mammals, the balance between copper necessity and toxicity is achieved at both cellular level as well as tissue and organismal levels (Harris, 1991; Bull and Cox, 1994).

##### Slc31a1 (Ctr1)

*Slc31a1* (Ctr1) was suggested to be the primary avenue for copper uptake in mammalian cells (Kuo et al., 2001). Ctr1 is highly expressed in the mouse CP during development and in adult mice (Kuo et al., 2001, 2006; Dahlin et al., 2009). Copper deficiency enhanced Ctr1 expression in the mouse and rat CP (Prohaska et al., 2003; Gybina and Prohaska, 2006; Kuo et al., 2006), suggesting an attempt to sequester limiting copper from the CSF (Gybina and Prohaska, 2006). The generation of Ctr1 knockout mice supports the importance of Ctr1 in brain function (Kuo et al., 2001; Lee et al., 2001). Early embryonic lethality was observed in homozygous mutant embryos and a deficiency in copper uptake was observed in the brains of heterozygous animals (Kuo et al., 2001). Ctr1 homozygous embryos could be recovered at E8.5 but were severely developmentally retarded and morphologically abnormal (Kuo et al., 2001). Copper was reduced to approximately half in the brains of heterozygous adult mice compared with control littermates, implicating Ctr1 is essential for copper entry into the brain (Kuo et al., 2001). The activity of the copper-dependent enzymes cytochrome *c* oxidase (CCO) and Cu, Zn-superoxide dismutase (Sod1) were also affected in the heterozygous mice (Lee et al., 2001). This reconfirmed older dietary data suggesting the importance of copper for normal mammalian development.

#### Zinc transporters

Zinc is required as a catalytic cofactor for many enzymes and it is important for protein domain structure stabilization (Devirgiliis et al., 2007). It is an essential component for the catalytic and structural activities of a number of secretory, membrane-bound, and endosome/lysosome-resident enzymes and thus plays important roles in a number of secretory processes (Coleman, 1992; Vallee and Falchuk, 1993). Zinc also acts as a signaling molecule and has been implicated in synaptic transmission (Takeda, 2000; Frederickson et al., 2005; Sensi et al., 2009). In the CNS, zinc plays a role in myelination by stabilizing interactions between lipids and myelin basic protein (Earl et al., 1988; Nuzzo et al., 2002). Mild zinc deficiency impairs cognitive development in rats and it might also have similar effect in humans (Massaro et al., 1982; Keller et al., 2000; Bhatnagar and Taneja, 2001). On the other hand, zinc can mediate brain toxicity as shown in experimental brain trauma and ischemic models in rats (Suh et al., 2000a; Lee et al., 2002). The involvement of zinc in AD was also suggested as elevated levels of this metal have been measured in plaques in AD, and *in vitro* zinc is able to promote β-amyloid aggregation (Lee et al., 1999; Suh et al., 2000b; Bush, 2002).

Because zinc is both essential and potentially toxic to the brain, mechanisms are needed to regulate its homeostasis. This is accomplished predominantly by members of two families of zinc transporters, the Slc30 family which mediates zinc efflux, and the Slc39 family which mediates zinc influx (Belloni-Olivi et al., 2009). Among the zinc transporters from the *Slc30* and *Slc39* families, *Slc39a4* (Zip4) was found to be expressed at a high level in the mouse CP by our ABA expression profiling study (supplemental Figure 1 from Dahlin et al., 2009). *Slc39a4* has also been detected in the rat CP along with *Slc39a1* (Zip1; Belloni-Olivi et al., 2009). In our ABA expression profiling study, *Slc39a1* is expressed at a low level in the mouse CP (supplemental Figure 4 from Dahlin et al., 2009).

##### Slc39a4 (ZIP4)

ZIP4 appears to transport a narrow range of metals (Dufner-Beattie et al., 2003a). Currently, there is no data on the function of ZIP4 in the CP. ZIP4 mRNA is most abundantly expressed in tissues involved in zinc homeostasis, such as the intestine, visceral endoderm, pancreatic islets, and yolk sac (Dufner-Beattie et al., 2004; Belloni-Olivi et al., 2009). Several members of the *Slc39* family respond to changes in extracellular zinc at the protein level, but Zip4 is the only member whose level of mRNA and protein increases in mouse tissues such as intestines and embryonic visceral yolk sac (Dufner-Beattie et al., 2003b). Because ZIP4 levels are regulated by dietary zinc, it was suggested that the brain has the potential to adapt to changes in zinc status (Belloni-Olivi et al., 2009). Embryonic lethality was observed in homozygote Zip4 mice while severe neuro-developmental problems were seen in heterozygous Zip4 knockout mice (Dufner-Beattie et al., 2007; Andrews, 2008). Heterozygous Zip4 knockout mice were also hydrocephalic (Dufner-Beattie et al., 2007; Andrews, 2008), which may be due to abnormal accumulation of CSF in the ventricles. These studies suggest that Zip4 might be involved in zinc homeostasis in the brain and may play an important role in the mammalian CP.

##### Slc39a12

In addition to Zip4, our ABA expression profiling study identified high levels of expression of a putative zinc transporter, *Slc39a12*, in the mouse CP (Dahlin et al., 2009). Slc39a12 is assigned to the zinc transporter family Slc39 but whether zinc is a physiological substrate of Slc39a12 is not known at present. There is currently no known function of this transporter in the CP or other tissues, although an earlier study suggested it might be a susceptibility gene in schizophrenia (Bly, 2006). Clearly, more studies are needed to elucidate the substrate, localization, and functional role of Slc39a12 in the CP.

## Conclusion

In this review, we have discussed the localization and physiological significance of a number of Slc transporters in the mammalian CP based on data obtained from our recent *Slc* gene expression profiling study in the adult mouse brain (Dahlin et al., 2009). Our prior analysis revealed distinct expression patterns of CP *Slc* transcripts as compared to other brain structures and identified *Slc* genes with the highest expression levels in the CP (Dahlin et al., 2009). A review of these highly expressed *Slc* genes suggests critical roles of Slc transporters in CSF secretion, energy production, and transport of nutrients, hormones, neurotransmitters, sulfate, and metal ions at the BCSFB. We hope our study will provide a useful resource for investigators to explore the roles of *Slc* genes in physiological and pathological processes and stimulate further research interest in this area. It should be noted that at the time of our ABA expression profiling analysis, ISH data was only available for ∼82% of the known mouse *Slc* genes, therefore a significant number of *Slc* genes was not examined in our study. In addition, given the large number of *Slc* genes expressed in the CP, it is beyond the scope of this review to discuss each individual gene involved. While we chose to focus on the 28 *Slc* genes most highly expressed in the CP based on our ABA expression profiling study, this by no means suggests that *Slc* genes expressed at lower levels are not as important. In fact, many *Slc* genes previously shown to be important for CP function (e.g., those shown in Table 2) are not among the 28 most highly expressed *Slc* genes in the mouse CP we have identified. An example is the electrogenic sodium bicarbonate cotransporter Slc4a5 (NBCe2), which was not included in our ABA expression profiling study but is essential for normal CP function based on studies with genetic knockout mice (Kao et al., 2011). In addition, our ABA expression profiling study was based on ISH analysis of mRNA expression levels, which may not faithfully reflect protein expression and did not account for the influences of posttranslational regulation. For instance, Slc transporters with slower turnover rates could have higher protein expression/activities than would be expected from their mRNA levels.

While a considerable amount of studies have been performed to elucidate the molecular mechanisms of ion transport and CSF production in the CP (Brown et al., 2004; Praetorius, 2007; Damkier et al., 2010; Majumdar and Bevensee, 2010), our knowledge regarding the specific function of various Slc transporters, such as those involved in energy balance, metabolism, signaling, nutrient, and xenobiotic transport at the BCSFB, is still rather limited. Our previous expression profiling study identified a large number of *Slc* genes expressed in the mouse CP (Dahlin et al., 2009). Detailed characterization of the encoded transporters, including the elucidation of their substrate specificities, transport mechanisms, and membrane localization (apical vs. basolateral) in the CP represent the very first steps in understanding their physiological functions at the BCSFB. In addition, *ex vivo* transport assays and generation of animal models such as CP-specific gene knockout mice by the Cre-recombinase/loxP system (Crouthamel et al., 2012) along with the use of specific inhibitors or antisense knockdown methods could be used to study the *in vivo* functions of these transporters. These studies will provide unique insights into the specific roles Slc transporters play at the BCSFB and will reveal the significance of these transporters in sustaining the normal functions of the CP and the CNS.

It should be pointed out that although the focus of this review is on SLC/Slc transporters expressed in the CP, members of both the ATP-binding cassette (ABC) and SLC transporter superfamilies may act in concert to mediate solute transport at the BCSFB. Therefore further knowledge of both of these transporter superfamilies will be required for a comprehensive understanding of the transport mechanisms at the BCSFB. In addition, although the BBB and the BCSFB are two distinct barrier systems, both of them play a role in the supply of important ingredients for brain function and homeostasis as well as protect the brain from circulating xenobiotics and blood-borne pathogens. It is interesting that data from our previous study showed a few *Slc* genes, including the thyroid hormone transporters *Slc16a2* and *Slco1c1*, the sulfate transporter *Slc13a4*, the reduced folate transporter *Slc19a1*, the l-ascorbic acid transporter *Slc23a2*, the proline transporter *Slc6a20*, and the system N amino acid transporter *Slc38a3*, are all co-expressed at high levels in both the brain microvessels and the CP (Dahlin et al., 2009). Transporters encoded by these *Slc* genes may play coordinated roles at the two blood-CNS interfaces to regulate brain homeostasis of nutrients and hormones and protect the brain from xenobiotics and toxins. Additional expression and functional studies are needed to further characterize ABC and SLC proteins in the mammalian BBB and BCSFB. Knowledge from these studies will help us understand how the brain interacts with the rest of the body through the two well-known but still under-studied brain barriers.

## Conflict of Interest Statement

The authors declare that the research was conducted in the absence of any commercial or financial relationships that could be construed as a potential conflict of interest.

## Footnotes

^1^http://www.bioparadigms.org/slc/menu.asp

^2^http://mouse.brain-map.org
